# Additive Manufactured Piezoelectric-Driven Miniature Gripper

**DOI:** 10.3390/mi14040727

**Published:** 2023-03-25

**Authors:** C. Andres Ferrara-Bello, Margarita Tecpoyotl-Torres, S. Fernanda Rodriguez-Fuentes

**Affiliations:** 1 Posgrado en Ingeniería y Ciencias Aplicadas del Instituto de Investigación en Ciencias Básicas y Aplicadas-Centro de Investigación en Ingeniería y Ciencias Aplicadas (IICBA-CIICAp), Universidad Autónoma del Estado de Morelos (UAEM), Cuernavaca 62209, Mor., Mexico; carlos.ferrarabll@uaem.edu.mx; 2 Centro de Investigación en Ingeniería y Ciencias Aplicadas (CIICAp), Universidad Autónoma del Estado de Morelos (UAEM), Cuernavaca 62209, Mor., Mexico; 3 Posgrado en Sustentabilidad Energética del Instituto de Investigación en Ciencias Básicas y Aplicadas-Centro de Investigación en Ingeniería y Ciencias Aplicadas (IICBA-CIICAp), Universidad Autónoma del Estado de Morelos (UAEM), Cuernavaca 62209, Mor., Mexico; sahiril.rodriguez@uaem.edu.mx

**Keywords:** micromanipulation, PLA, piezoelectric actuation, microdisplacement

## Abstract

In several cases, it is desirable to have prototypes of low-cost fabrication and adequate performance. In academic laboratories and industries, miniature and microgrippers can be very useful for observations and the analysis of small objects. Piezoelectrically actuated microgrippers, commonly fabricated with aluminum, and with micrometer stroke or displacement, have been considered as Microelectromechanical Systems (MEMS). Recently, additive manufacture using several polymers has also been used for the fabrication of miniature grippers. This work focuses on the design of a piezoelectric-driven miniature gripper, additive manufactured with polylactic acid (PLA), which was modeled using a pseudo rigid body model (PRBM). It was also numerically and experimentally characterized with an acceptable level of approximation. The piezoelectric stack is composed of widely available buzzers. The aperture between the jaws allows it to hold objects with diameters lower than 500 μm, and weights lower than 1.4 g, such as the strands of some plants, salt grains, metal wires, etc. The novelty of this work is given by the miniature gripper’s simple design, as well as the low-cost of the materials and the fabrication process used. In addition, the initial aperture of the jaws can be adjusted, by adhering the metal tips in the required position.

## 1. Introduction

Micro-/nano-manipulation systems can achieve different functions, such as the gripping, decoupling, puncturing, injecting, and positioning of small objects with micro/nanometric dimensions [1]. Their operation can be manual or automated, and their size should not necessarily have dimensions in the order of the samples to be manipulated. Micromanipulation systems have achieved great relevance due to the need to manipulate small-sized clamping objects, which is required in several areas, with controlled conditions of force, displacement, and time. 

Micromanipulation systems are widely used in different research and industrial areas due to the miniaturization trend, the sophistication of microelectromechanical systems (MEMS), and in general, technology advancement [2]. These systems have applications in biomedicine [3], microassembly [4], nanomedicine, and biology [5], among others. For example, in the domain of cell biology, a micropositioning system can be used to move a microinjection pipette to transfer drugs [6]. In the area of MEMS, microassembly is often used to simplify manufacturing processes when designs are complex or different micromanufacturing technologies are used [4].

The structure of the micromanipulation systems is composed of different elements, usually a control system, a displacement platform, a tool for holding the sample, and a microscopic visual servo drive system, as well as various displacement and force sensors [7]. The sliding platform and the clamping tool are mechanical elements that can be manufactured with compliant [8] or rigid [9] body mechanisms. There is an increasing trend to create designs with compliant mechanisms due to their advantages over rigid body ones, because being structures manufactured in one piece, they are lubricant-free, have no friction losses, no backlash, and mass production is easily facilitated, and there are other advantages, such as high precision and low weight [10]. 

Microgrippers with one degree of freedom (DOF) have an opening/closing movement of their fingers. With two DOFs, they can grip, hold, and release an object; in this case, the two fingers are controlled independently. In [11], a microgripper with two DOFs and the development of a platform useful for researchers in micromanipulation are shown and tested. In [12], a microgripper with multi DOFs is reported, whose fingers can open/close and have up/down movements, increasing its functionality.

Microdisplacement platforms with flexible mechanisms that can move the sample, while the clamping tool remains static, have been reported in the scientific literature and have been designed with two- [13], and three- [14] axis movement, as well as three-axis and angular movements [15]. It is convenient that the microdisplacement platforms are designed with multiple DOFs, because they can manipulate samples with irregular geometries; however, the complexity of the designs also increases.

On the other hand, different microhandling options are available on the clamping tool, which can be classified as contact-free and contact handling [16]. Examples of non-contact manipulators are optical [17], acoustic [18], dielectrophoretic [19], and magnetic [20] grippers. The advantage of contact-free methods lies in their precision and non-invasiveness, which is the reason for their wide use in the manipulation of cells, nuclei, and other biological specimens in clinical and biomedical areas [21]. Contact manipulation techniques use a tool that grips the object using one of the following principles: material closure, gripping by traction, and by form-fitting, in accordance with [22]. 

Mechanical microgrippers with a wide variety of designs and actuation methods are part of the clamping by traction group; they are very popular for 3D solid object micromanipulation applications and can be manufactured with micrometer or even millimeter dimensions, but the motion control in the jaws must be able to generate micrometer or nanometer displacement steps. Unlike non-contact grippers, with one DOF on the jaws, sufficient force can be exerted to deform the object to be manipulated; this is a problem when this deformation is not desired. If the exerted force is not enough, the sample may become detached. Among the solutions to control the force level, the design of multi-finger grippers [23], with piezoresistive cantilever beams to monitor the contact force in the jaws, is found in [24].

Generally, mechanical microgrippers are manufactured in one piece with compliant mechanisms of an extruded 2D design. It is also possible to implement microgrippers with more complex 3D designs, which can be assembled by more than one piece with flexible mechanisms [25] or fabricated by any additive manufacturing technique [26,27]. For microgrippers with micrometer dimensions, micromanufacturing processes, commonly used in MEMS, are used. Millimeter-scale grippers can be manufactured using CNC machining, wire electrical discharge machining (WEDM), additive manufacturing, using conventional 3D printers [28], and PolyJet printing processes [29], among others. It is worth mentioning that the processes used for the development of MEMS are costly in terms of prototype manufacturing, being significantly reduced when mass production is carried out. To validate the performance of our design, additive manufacturing was chosen, which is convenient because of its affordability.

Regarding the actuation methods, thermal, electrothermal, electromagnetic, electrostatic, piezoelectric, shape memory alloys (SMA), and fluidic actuation have been widely reported in the case of microgrippers manufactured with MEMS technology. In the case of miniature grippers with millimeter dimensions, the number of actuation methods decrease, because the mechanisms of the miniature grippers require more force. SMA, electrothermal, and piezoelectric actuation processes are suitable for microgrippers at this scale [30]. Piezoelectric actuation is preferred due to its high force output to weight ratio, fast response, zero backlash [31], and nanometer displacement resolution. Table 1 shows a comparison between different piezoelectric-driven microgrippers made of aluminum. 

It should be noted that no piezoelectrically actuated miniature grippers made by additive manufacturing with polylactic acid (PLA) were found in the scientific literature. However, 3D printed microgrippers with other forms of actuation were found; for example, a microgripper made with CN982A75 and magnetically actuated was reported in [23]. Another microgripper made with PLA, which is mechanically controlled by a motor with wires attached, which allows the opening/closing of the jaws, is shown in [31,32]. Grippers [33] and microgripper [34,35] made with other polymers have also been reported. 

This article shows the development of a micromanipulation system composed of a Miniature Gripper (MG) for micro-objectives clamping, and a semi-automatic control system. The miniature gripper, designed with PLA, has a set of compliant mechanisms that amplify the input movement, allowing the jaws to be moved. A piezoelectric actuator has been developed, consisting of an array of stacked buzzers capable of generating an unloaded linear displacement of approximately 360 µm, which outperforms most commercial actuators at a lower cost. This miniature gripper can be integrated with the platform previously reported [8] to increase the micromanipulation system capabilities.

This work focuses on the development of the MG, which is one of the most important devices of the micropositioning system, because the adequate manipulation of micro-objects depends on it. The contribution of this work is to propose an affordable piezoelectric micromanipulation system, with enough precision to be used in research tasks when microfabrication is not an achievable alternative. It could also be useful for didactic purposes, including it in activities to increase the motivation of vocations in STEM (Science, Technology, Engineering, and Mathematics).

**Table 1:** Characteristics of microgrippers based on piezoelectric actuation.

Ref.	Material	Amplification Factor	Maximum Displacement (µm)	Output Force (mN)	Frequency (Hz)
[36]	AL7075T651	15.5	134	N.A.	N.A.
[37]	AL7075T651	16.4	150.8	1870	N.A.
[38]	AL7075T651	13.94	121	50	N.A.
[12]	AL7075T651	22.8	190	1000	N.A.
[39]	AL7075	21.4	427.8	50	N.A.
[40]	7075-T6	8.1	154	N.A.	N.A.
[41]	Mn65 and Al-7075	52.8	918	50	217.2
[42] *	AL7075	31.6	≈700	N.A.	N.A.
[43]	Al alloy	16.8	102.3	227.7	N.A.
[44] *	AL7075	20.82	236.68	366.7	746.56

NOTES: * These systems involve two piezoelectric actuators for the microgrippers actuation, instead of only one. N.A.: Not Available.

## 2. Materials and Methods

### 2.1. Materials

In this section, the implementation of the microgripper is shown. The structural support of the piezoelectric driver or actuator was manufactured using PLA-based additive manufacturing, which contributes to obtaining a low-cost prototype. In the previous design [45], the piezoelectric driver was composed of 4 buzzers electrically connected in parallel; in this case, the number of buzzers, with diameters of 27 mm, has been increased to 6, with larger diameters (35 mm). This fact allows us to increase the displacement by approximately 121%. The use of these low-cost and high availability buzzers also contributes to reducing the overall cost of the MG implementation.

In the preliminary design, only the tips of the jaws were made using recycled aluminum, obtained from a hard disk, and manually cut. Once its operation was tested, the fabrication of the current prototype was made. The tips were manufactured with structural aluminum, with a thickness of 0.4 mm, using a numerical control machine (CNC). It should be noted that any other type of aluminum, of equal or similar thickness, could be useful; it is even possible to use needle tips, previously polished. 

### 2.2. Methods

Based on the Pseudo Rigid Body Model (PRBM), the appropriate length of the parallelogram mechanism of the MG was determined to obtain the desired amplification factor. The MG performance parameters were validated with ANSYS^®^. The smallest elements are the flexure hinges, whose dimensions were determined by also considering the precision of the manufacturing process, which is limited by the diameter of the printer nozzle, in this case 0.2 mm. It should be noted that, when the manufacturing process is carried out, there is an additional thickening of approximately 0.2 mm over the minimum width to be engraved with the nozzle. A flexure hinge width of 0.5 mm was designed; however, its fabricated size was 0.7 mm. Due to the burrs on the edges of the elements, it was necessary to rough them using a driller with a slim drill width, for approximately 20 min, obtaining a width of the flexure hinges of 0.5 mm, as planned.

Ultimaker Cura© (version 4.1.0) was used for managing the printing process and changing some printer properties. Table 2 shows the suggested parameter settings for the printing process using PLA. The ANET A8 3D printer was used. The Build Plate Temperature Range was recommended to enhance the adhesion of PLA to the plate. At these temperatures, a skirt is appropriate to begin the printing process. In the case of low adhesion or long printing time, brim could be the option.

**Figure 1:**
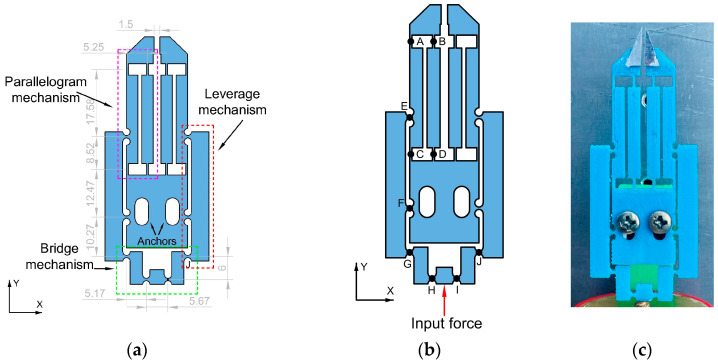
(**a**) Schematic diagram of MG based on compliance mechanisms. Dimensions are in millimeters. (**b**) Schematic diagram for functional description. (**c**) Photograph of the MG with metal tips added, with the initial aperture adjusted to achieve a final aperture of 1.5 mm, enough to perform practical tests.

The geometry of the proposed MG includes, for the transmission of equal displacement to each lever amplifier, a bridge-type mechanism. The leverage mechanism on each clamping arm is used for displacement amplification. Finally, for precise opening/closing of the jaws, a parallelogram mechanism is added (see Figure 1).

The proposed MG can be complemented with the micropositioner reported in [8], also made with recycled or, alternatively, low-cost materials. This assembled system would provide a complete micromanipulation system for research or academic purposes.

## 3. Miniature Gripper Design Description

The MG was designed considering a buzzers stack actuator that delivers a lower locking force (≈2 N) than commercial piezoelectric actuators, which can provide up to a few thousand N [46,47]. The limited actuator force affected the total amplification factor (*Amp*) of the MG, which corresponds to the sum of the individual displacement of its jaws. After MG modelling, a factor closer to 12 is obtained, which can provide a useful output displacement.

The design of the MG with symmetrical geometry was chosen to obtain a larger displacement between the jaws, because an asymmetrical design may have less output displacement [42]. In addition, if there is a fracture of a flexible element, e.g., part of one of the jaws, the MG could continue to operate temporarily, with a smaller displacement at the output. 

Rectangular and circular flexure hinges were used in the compliant mechanisms. The first one is more suitable for a wide range of rotation and low stiffness, while the last one is suitable for a smaller range of rotation and higher stiffness [48].

The schematic design of the MG is shown in Figure 1; it basically has:

- Three groups of compliant elements:
  1. Bridge mechanism: This is the input amplification stage, where the force of the piezoelectric stack is applied. It is formed by flexure circular hinges, GHIJ. The motion received and transmitted is moderately amplified.
  2. Leverage mechanisms GFE: They are located at the middle stage and have flexure circular hinges. They move the correspondent parallelogram mechanism though joint E, achieving the jaw displacement.
  3. Parallelogram mechanisms ABCD are the last amplification stages of the MG design. They have the largest rotational movements in their rectangular hinges. Jaws are at the top of each parallelogram mechanism.
- MG’s jaws. Modifications of the previous model [45] were made to improve its performance. The shape of the handmade tips was modified and machined with a CNC.
- The anchor: constitutes the fixed part of the MG, where the oval holes allow fixture using screws.

With this design, the movement is transmitted from the bridge mechanism to the MG’s jaws. Figure 1b shows details of the functionality of the system. This arrangement focusses the displacement on the X-axis (when jaw aperture or closing is performed). The parallel movement of the microgripper jaws ensures precise and reliable clamping of the samples [49].

The mechanical anchors define the aperture of the normally open MG’s jaws in their initial state. The anchor holes serve for fixing the bridge mechanism close to the tip of the piezoelectric stack, creating a pre-load, which serves to reset the piezoelectric stack to its initial position (when the piezoelectric actuator is off, the maximum aperture of the MG’s jaws is given).

A drawback of MG implementation in PLA is the high roughness of the surfaces. These imperfections are especially important in the lateral walls of the jaws, providing a non-uniform closing. A simple solution is to perform micro-polishing of the jaws’ walls, improving their performance in object micromanipulation and avoiding causing damages. However, this is not an easy task, because it must be done without reaching a temperature that deforms the jaws. 

Aluminum tips were added to the MG ones made of PLA to increase their uniformity. They have a right-angle triangle shape, with a thickness of 0.4 mm. The adjacent and opposite sides’ lengths are 4 mm and 10 mm, respectively, and the hypotenuse is 10.77 mm. They were manufactured with a CNC. Another alternative to increase the accuracy is to construct interchangeable tips by photolithographic techniques [40], or others. This adaptation is also shown in Figure 1c, which shows the location of the tips that were manually attached to the horizontal reference from the point where the width of the PLA-printed tip starts to decrease. An industrial microscope was used to increase the glue process precision. Cyanoacrylate combined with sodium bicarbonate (NaHCO_3_) was used to reduce drying time. A uniform force was applied to keep the clamp opening in the desired position during the bonding process.

The stiffness of the bonded metal tips is sufficiently high. This characteristic was validated because they were not deforming under applied loads, limited to 13.24 mN, according to the results of analytical and numerical calculations. In addition, they do not detach unintentionally either; if necessary, pressure must be applied with a sharp knife between the tips of both materials to achieve a point of support that allows them to be detached.

## 4. Analytical Model

### 4.1. Kinematic Modeling

**Figure 2:**
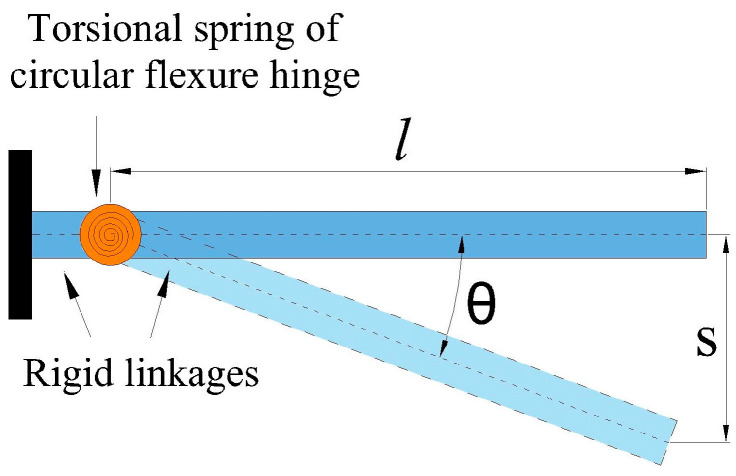
Equivalence of a circular flexure hinge to the PRBM.

The kinematic modeling of the MG was performed based on the PRBM method [50], which simplifies the analysis. In this method, the flexure hinges are considered as torsional springs and the connecting elements as rigid bodies. Figure 2 shows a circular flexible hinge and its representation under PRBM analysis.

According to Figure 2, when a force is exerted on the free end of the linkage, a rotational deformation occurs, where the rotation center is at the torsional spring and the radius of rotation corresponds to the length of the free linkage. If the deformation angle, *θ*, is very small, Equation (1) is satisfied.

(1) $$\theta =\tan^{-1}\left(\frac{s}{l}\right)\;≅\;\frac{s}{l}$$

**Figure 3:**
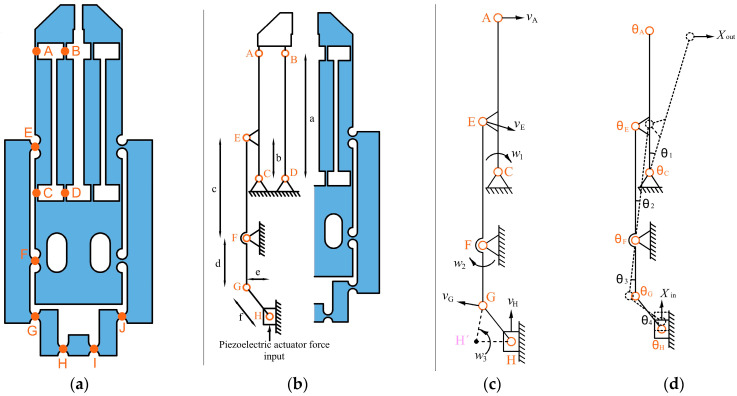
MG. (**a**) Schematic diagram. (**b**) PRBM and lengths of the linkage elements. (**c**) Angular and instantaneous velocities. (**d**) Rotational springs.

For the kinematic model, the stiffness of the flexure hinges is discarded, and they are considered as conventional bearings. Due to the symmetry of the MG (Figure 3a), only half of the compliant mechanisms were analyzed; therefore, the simplified structure consists of eight bearings, denoted by j (j = A, B, ..., H) and four linkage elements, as shown in Figure 3b. In Table 3, the dimensions of the MG elements are given.

To obtain the *Amp* of the MG (Figure 3a), the instantaneous velocities at points A, E, G, and H shown in Figure 3c were calculated. Each one is obtained by setting the rotation center of the linkage, which is perpendicular to the direction of the linkage end velocity; likewise, the length of the linkage under analysis is set as the radius ($l_{IA}$, $l_{GF}$, $l_{GH’}$, $l_{EF}$, $l_{EC}$,$\;l_{AC}$) and the angular velocity is represented as ω. Therefore, the instantaneous velocities of the mentioned points are:

(2) $$\;\;\;\;\;\;\;\;\;v_{H}=\omega_{3}l_{H’H}$$

(3) $$\;\;\;\;\;\;\;\;\;v_{G}=\omega_{2}l_{FG}\;=\omega_{3}l_{GH’}$$

(4) $$\;\;\;\;\;\;\;\;\;v_{E}=\omega_{2}l_{EF}=\omega_{1}l_{EC}$$

(5) $$\;\;\;\;\;\;\;\;\;v_{A}=\omega_{1}l_{AC}$$

To calculate the total displacement amplification ratio, two points were considered: in the first one, the deformations of the flexure hinges are very small, and in the second one, similar length values $l_{H’H}≅l_{H’G}$ are taken (see Figure 3c). With these conditions, the following ratio is obtained:

(6) $$Amp=\frac{2X_{Out}}{X_{in}}=\frac{2v_{A}}{v_{H}}\;≅\frac{2ac}{bd}$$

where $X_{in}$ is the input displacement and $X_{Out}$ is the output one.

Therefore, *Amp* depends on the dimensions of the linkages of the leverage and parallelogram mechanisms. Substituting the values shown in Table 3, in Equation (6), the MG design has an individual *Amp* of 6.26 in each of the symmetrical parts of the MG; therefore, *Amp* has a value of 12.52, which is larger than the corresponding value (6.5) of the MG shown in [45].

Additionally, it is necessary to calculate the rotational deformation of the torsional springs for the static analysis (Figure 3d); taking the same conditions to calculate the amplification ratio, the rotational angles ($\theta_{A},\ldots ,\theta_{H}$) satisfy the following equations:

(7) $$\theta_{H}=\;\theta_{G}=\theta_{4}=\frac{X_{in}}{e}$$

(8) $$\theta_{F}=\theta_{2}=\theta_{3}$$

(9) $$\;\theta_{2}=\frac{\frac{AmpX_{in}}{2}}{\frac{ac}{b}}=\frac{X_{in}}{d}$$

(10) $$\;\;\theta_{A}=\theta_{B}=\theta_{C}=\theta_{D}=\theta_{1}=\frac{AmpX_{in}}{2a}=\frac{cX_{in}}{bd}$$

(11) $$\theta_{E}=\theta_{C}+\theta_{F}$$

In Equations (7), (9), and (10), it was assumed that the angles *θ ≈ 0*, then *tanθ ≈ θ.*

### 4.2. Static Modeling

**Figure 4:**
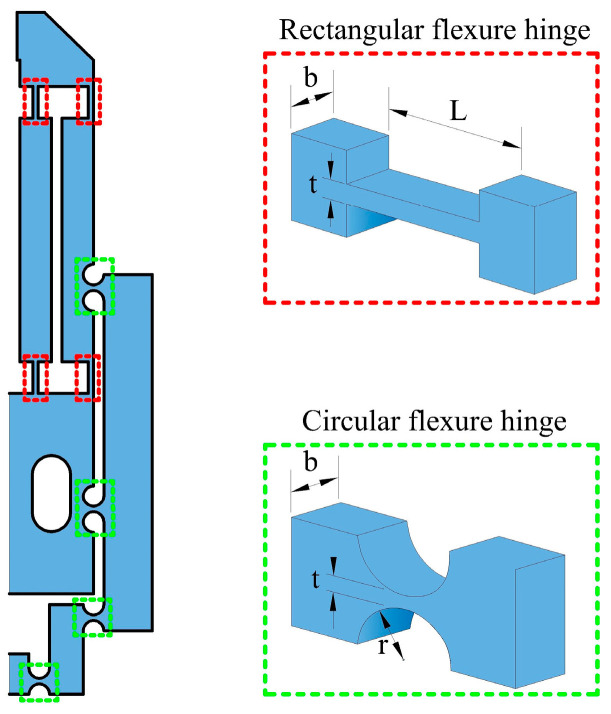
Rectangular and circular flexible hinges implemented in the MG design.

The rotational stiffness of rectangular flexure hinges (Figure 4) can be calculated by [48]:

(12) $$K_{i}=\frac{\mathrm{Eb}t_{i}^{3}}{12L}\;\;\;\;\;\;\;\;\;\;\;i=A,B,C,D$$

For circular flexure hinges (Figure 4), the rotational stiffness is given by [48]:

(13) $$K_{i}=\frac{2\mathrm{Eb}t_{i}^{5/2}}{9\pi r_{i}^{1/2}}\;\;\;\;\;\;\;\;\;i=E,F,G,H$$

where *E* = 3.56 Pa, *r* = 1 mm, *t* = 0.5 mm, L = 3 mm, and *b* = 1.5 mm. The stiffness values obtained from Equations (12) and (13), are 0.065648 Pa · m^3^ and 0.018229 Pa · m^3^, respectively. As can be observed, the rectangular flexure hinge is more flexible than the circular one, as expected.

On the other hand, the individual torque of each flexural hinge is:

(14) $$m_{i}=-K_{i}\theta_{i}{}_{}$$

The total system virtual work is given by:

(15) $${\delta W}_{sys}={\vec{F}}_{in}\cdot {\delta \vec{x}}_{in}-{\vec{F}}_{out}\cdot {\delta \vec{x}}_{out}+\underset{i}{\sum }{\vec{m}}_{i}\cdot {\vec{\delta \theta }}_{i}=0$$

Therefore:

(16) $$F_{in}=\frac{F_{out}x_{out}}{x_{in}}-\frac{\left(-K_{A}\theta_{A}^{2}-K_{B}\theta_{B}^{2}-K_{C}\theta_{C}^{2}-\cdots -K_{H}\theta_{H}^{2}\right)}{x_{in}}$$

Substituting the angle values and the stiffness constants in expression (16), the equation of $F_{in}$ is obtained.

(17) $$F_{in}=\frac{F_{out}Amp}{2}+\left[\frac{c^{2}K_{A}+c^{2}K_{B}+c^{2}K_{C}+c^{2}K_{D}+c^{2}K_{E}}{{\left(bd\right)}^{2}}+\frac{K_{E}+K_{F}}{d^{2}}+\frac{K_{G}+K_{H}}{e^{2}}\right]x_{in}$$

This force could be useful to calculate the output displacement. 

## 5. Finite Element Analysis

### 5.1. Main Parameters Determination

A Finite Element Analysis (FEA) was performed for the MG, specifically to determine the jaw displacement, its *Amp*, and the stress in the flexure hinges when different magnitudes of input force are applied. The mathematical model of the MG, calculated in the previous section, was also verified with this analysis, using the ANSYS Workbench™ tool. The PLA data used to perform this analysis are given in Table 4.

**Figure 5:**
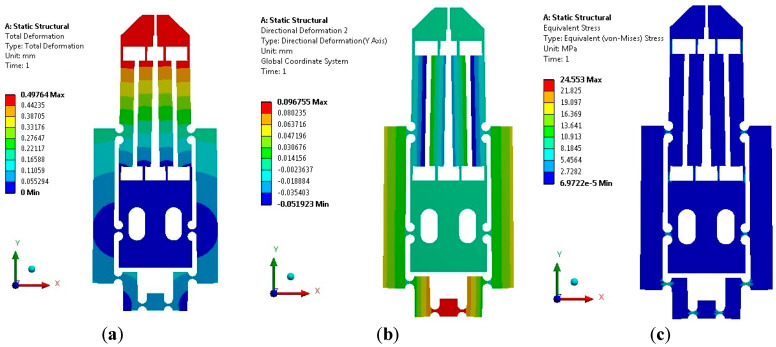
Performance of the MG when an input force of 2.5 N is applied. (**a**) Total and (**b**) directional deformation in Y-axis. (**c**) Equivalent stress distribution.

The analysis consisted of simulating the application of input force to MG, from 0.1 N, up to 2.5 N, with 0.1 N increments. For each input force value, the magnitude of the displacement in the jaws and the maximum stress of the MG was recorded. The simulation results are shown in Figure 5. The directional deformation of the MG in the X-axis, with the largest applied input force of 2.5 N, is shown in Figure 5a, where each jaw displaces 497.64 µm, with a maximum stress reached of 24.55 MPa. With the minimum applied force of 0.1 N, the displacement in each jaw was 19.9 µm and the maximum stress was 0.98 MPa.

Figure 5b shows the directional deformation in the Y-axis. The maximum displacement corresponds to the bridge mechanism; the movement between jaws is negligible. Therefore, the displacement of the jaws is mainly in the X-axis, which confirms the functionality of the parallelogram mechanism.

The maximum input displacement of the MG in the Y-axis is 96.75 µm, when the input force of 2.5 N is applied. Therefore, knowing the displacement of one jaw and the corresponding input displacement, the individual amplification factor of the MG can be calculated, which is 5.14 for each jaw. Because, theoretically, this factor is 6.26, then the error is 17.89%.

Figure 5c shows the stress distribution obtained when the MG is fed; the maximum stress (24.55 MPa) is located at the flexure hinges, as expected. This value is lower than the ultimate tensile strength for PLA (shown in Table 4). 

From simulation results, it is possible to observe that, under these conditions, the MG’s jaws have an appropriate in-plane aperture for manipulating objects over a wide range of sizes. 

In addition, it is verified that the dimension of the flexure hinges is adequate, including the thinner parts, which are also within the limits of stress permissible values according to Tensile Yield Strength (Table 4); this is also valid for the case when the maximum actuator force and displacement are applied.

**Figure 6:**
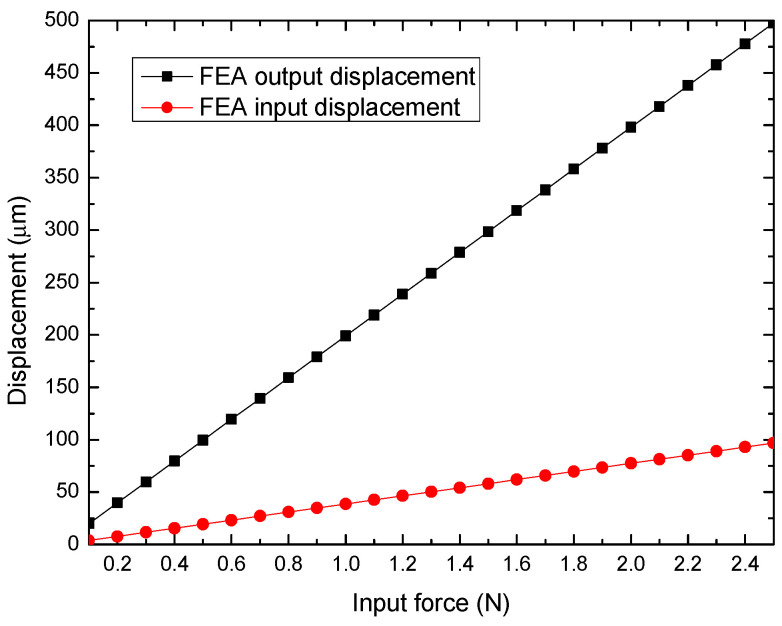
Input (at bridge mechanism) and output displacement (in each jaw) of the MG, under a force swept.

In Figure 6, the displacements at the input stage (bridge mechanism) and output of the MG (between jaws) are observed considering a sweep of input force applied from 0.1 N up to 2.5 N, with steps of 0.1 N. A linear behavior is observed in both cases, as well as the constant gain ratio (amplification factor) between both displacements.

### 5.2. Modal Analysis

**Figure 7:**
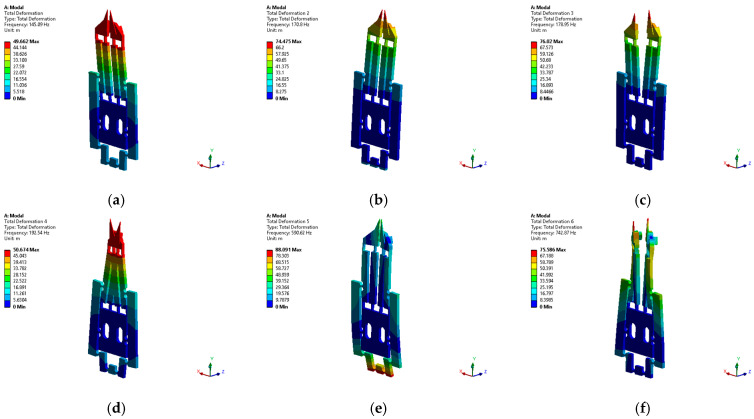
(**a**) First, (**b**) second, (**c**) third, (**d**) fourth, (**e**) fifth, and (**f**) sixth MG modal shapes.

The modal shapes are shown in Figure 7. Modal frequency 4 corresponds to the behavior of the normally open MG. As is known, in most symmetrical grippers, modal frequencies are very close [53]; a similar trend is observed here.

From the obtained results and considering that the maximum operating frequency, in practice, should be less than two-thirds of the first-order natural frequency, according to [54], in our case, the operating range is from 0 up to 96.7 Hz.

To smooth the surface of the MG jaws, its pins were replaced by metallic jaws, which allowed a uniform clamping. For tiny targets of dimensions close to the size of the tips, it was observed that there are no adhesion problems that prevent the object’s release. For much smaller targets, the release process can be facilitated by vibration (an active method), where vibration can be performed by applying a small voltage in AC, with a frequency much lower than the first natural frequency.

In Table 5, technical details of the numeric analysis are shown.

## 6. Experimental Setup and Results

### 6.1. Piezoelectric Stack Characterization

The piezoelectric actuator was designed as an array of 6 buzzers, 35 mm in diameter, connected in parallel and assembled on a support structure made in PLA; details of its construction can be found in [8].

The displacement of the piezoelectric stack without load was tested with a voltage source ranging from 0 V up to 110 V, with steps of 10 V. The limit of the voltage values was established due to the sensor saturation for higher levels. The device used to measure the displacement generated by the piezoelectric stack is a KAMAN inductive proximity sensor, model SMU9000-15N-0001, based on the electromagnetic induction effect. 

**Figure 8:**
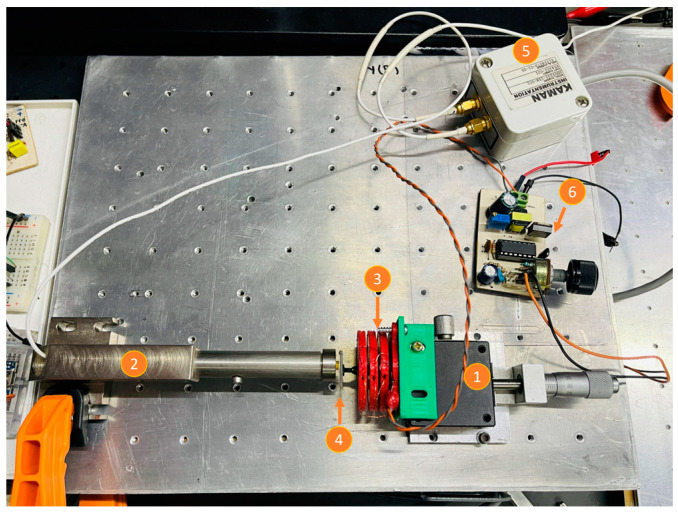
Experimental setup to measure the displacement generated by the piezoelectric stack. 1. Displacement platform. 2. Inductive sensor. 3. Piezoelectric stack. 4. Small square piece of reflective aluminum. 5. Control module. 6. Piezoelectric actuator driver.

**Figure 9:**
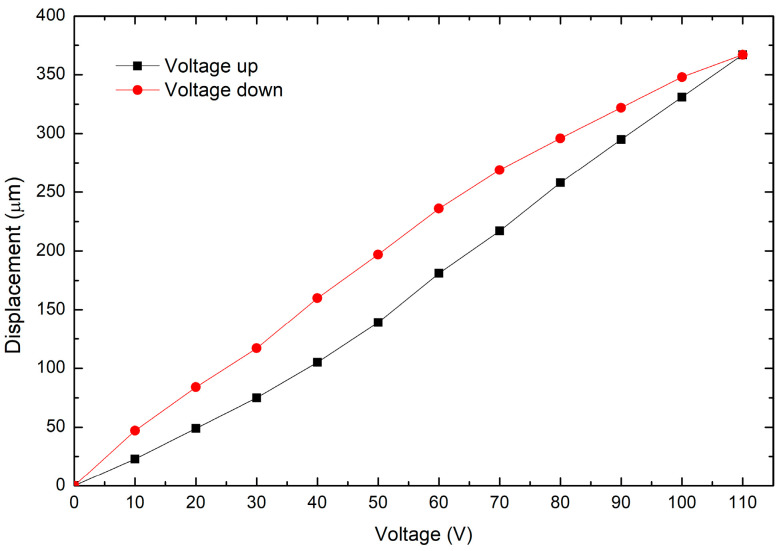
Displacement generated by the piezoelectric stack, without mechanical load.

The experimental setup consists of a microdisplacement platform, on which the piezoelectric actuator is horizontally fixed in front of the inductive sensor, as shown in Figure 8. By means of the microdisplacement platform, the actuator is slowly advanced towards the sensor, until it begins to detect the target. At the tip of the piezoelectric actuator, a small square piece of aluminum was placed to achieve a correct detection by the sensor, supported by this highly reflective piece. A maximum displacement of 367 µm is observed at 110 V (see Figure 9). The hysteresis effect, characteristic of piezoelectric materials, is shown in this figure. 

**Figure 10:**
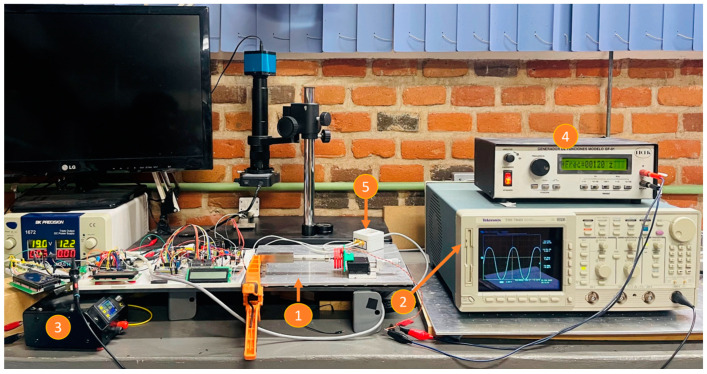
Experimental setup to determine the resonance frequency of the piezoelectric actuator. 1. Experimental setup (shown in Figure 8). 2. Oscilloscope Tektronix TDS 784D. 3. Power supply. 4. Frequency generator. 5. Control module.

**Figure 11:**
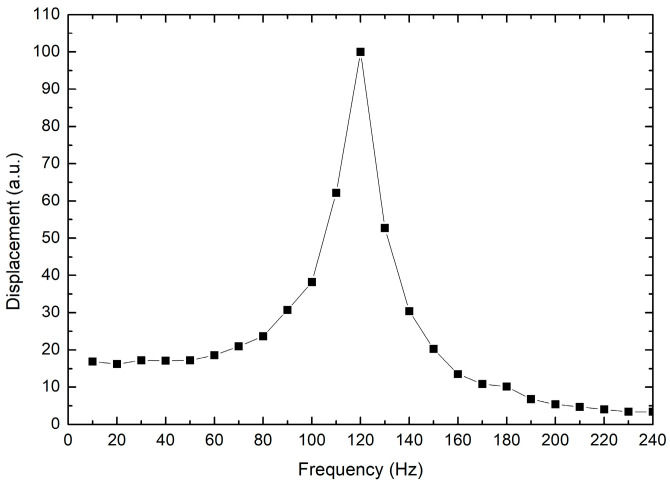
Displacement of the piezoelectric actuator when a frequency-varying sinusoidal signal with 20 V_pp_ is applied.

Another test was performed to find the resonance frequency of the piezoelectric actuator. An oscilloscope was connected to the output of the inductive sensor, and the power supply of the piezoelectric actuator was replaced by a function generator; the assembly of the actuator and the sensor was the same as previously described. The experimental setup for this test is shown in Figure 10. The procedure consisted of applying a sinusoidal signal (alternate current, AC), with a 20 V_pp_, to the piezoelectric actuator at different frequencies. The sweep was performed from 10 Hz up to 240 Hz, with steps of 10 Hz; in each of them the amplitude of the displacement was recorded by an oscilloscope. Figure 11 shows the obtained results. Even though the power supply is only 20 V_pp_, a maximum displacement was achieved at 120 Hz. This AC performance is important because a higher output displacement can be achieved than when a direct current (DC) voltage is applied. 

### 6.2. Characterization of the Amplification Ratio 

**Figure 12:**
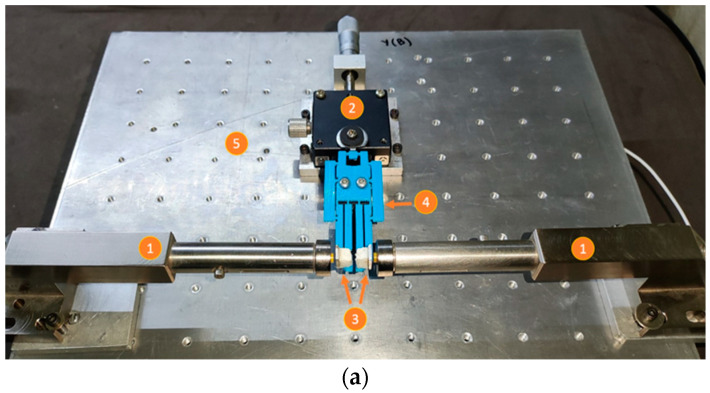

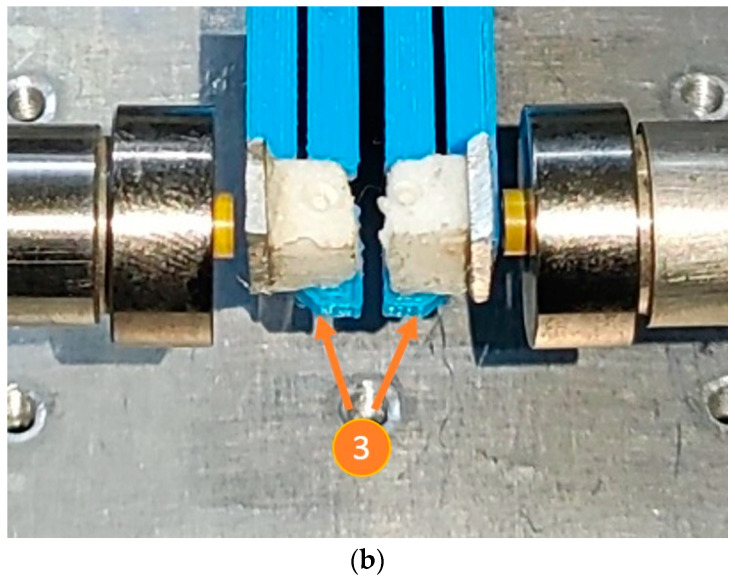
(**a**) Experimental setup for measurement of jaw displacement. (**b**) Zoom-in at the tips of the MG.

An experiment was carried out to determine the amplification factor of the MG, as well as to compare the experimental results with those obtained from the theoretical and FEA models, respectively. The experimental setup is composed of (Figure 12a):

1. Inductive sensors (to measure output displacements).
2. Scroll platform (to apply input displacements).
3. Small metal sheets. These surfaces were implemented with pieces of material taken from a hard disk drive (HDD) and placed at the test points, supported by dielectric material.
4. MG, additive manufactured with PLA.
5. Metallic base, made with Aluminum.

Figure 12b shows a zoom-in of the MG tips. The blank materials (dielectric of lightweight) are used as mechanical support for the metal scraps, which is necessary for the detection of the tip displacement by the inductive sensors. The additive manufacturing of the MG, using PLA, makes it necessary, for this test, for the metal scraps to be glued.

**Figure 13:**
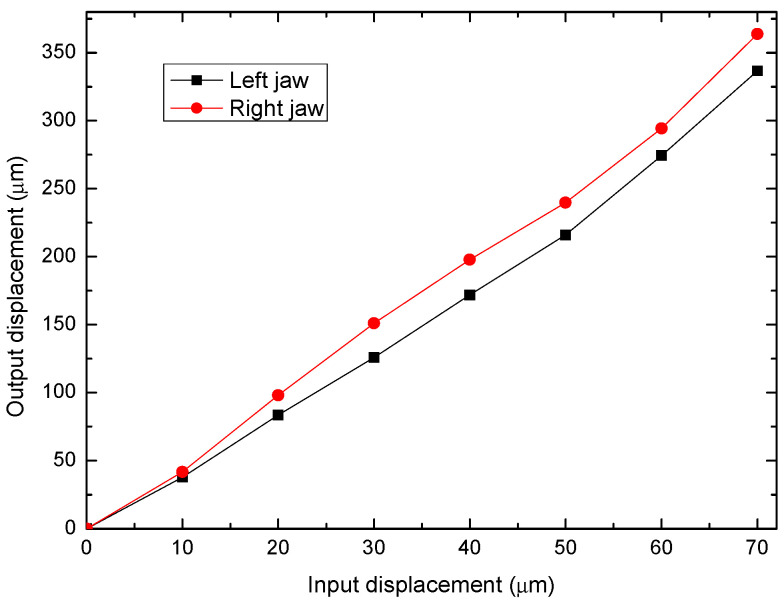
Output of MG jaw (or tip) displacement when an input displacement is applied.

The procedure consisted of applying displacements at the input of the MG, which corresponds to the bridge-type mechanism, in the range of 0 up to 70 μm, with increments of 10 μm. The displacement platform was used for this task. The incremented values obtained from the inductive sensors, corresponding to the output displacements of each jaw were recorded; see Figure 13. It was decided to limit the input displacement to this range, to maintain a wide safety margin for the flexible hinges, considering the stress value generated in them. According to the FEA results, for a displacement input value of 96 μm, the stress generated is equivalent to 41% of the tensile strength parameter; therefore, with a displacement of 70 μm the integrity of the flexure hinges is guaranteed.

The graph of the displacement in each jaw indicates a linear trend; however, there is a slight asymmetry. With an input displacement of 70 μm, the right jaw achieved a larger displacement, 363.7 μm, while the other one reached 336.6 μm, which is 7% less than the maximum displacement of the right jaw. The asymmetric displacement behavior can be attributed mainly to:

- The flexible mechanisms are not fully symmetrical. This fact could be provided by the manufacturing method (FDM) and the precision of the 3D printer, which was in-house designed.
- The motion input was not applied exactly at the middle part of the bridge-type flexible mechanism of the microgripper, because it was manually located.

Although this asymmetry of the movement did not cause any problems in the micro-object clamping tests, it is possible to correct it by adjusting the position of the small contact surface between the moving element (displacement platform) and the input of the MG (bridge-type mechanism), in the direction of the jaw with the least displacement. In the right jaw the minimum individual *Amp* was 4.16 and the maximum was 5.19, while in the left jaw the minimum value was 3.77 and the maximum was 4.8, with input displacements of 10 μm and 70 μm, respectively. 

**Figure 14:**
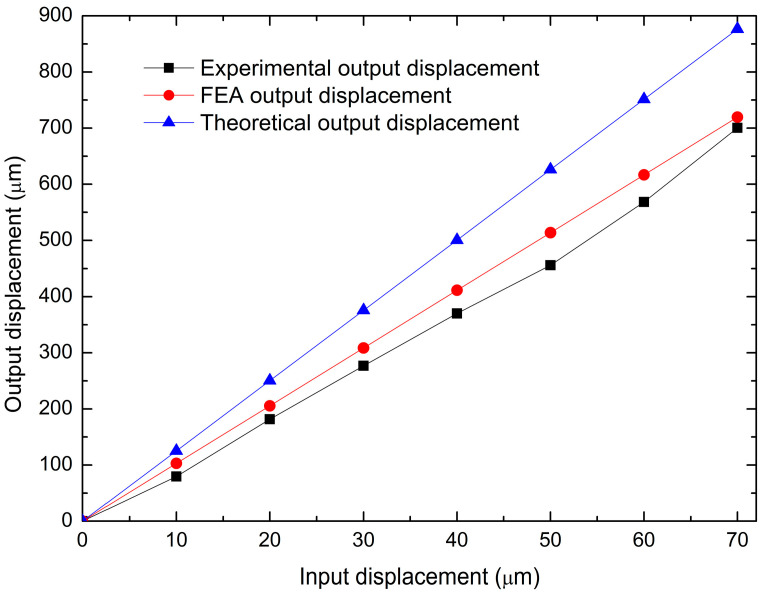
Comparison of the total amplification factor obtained experimentally, with the theoretical model and FEA.

Figure 14 shows a comparison of output displacement values obtained by the analyzed methods. As expected, the experimental results were lower than those obtained with the other ones, because, in the analytical model (PRBM), ideal conditions are considered, and in FEA, despite having greater precision in the calculations, and considering more parameters and conditions that make the result closer to reality, it is still a model with non-changing parameters and conditions, while in practice many variations are faced due to the semi-controlled environment.

### 6.3. Characterization of the Input Force

**Figure 15:**
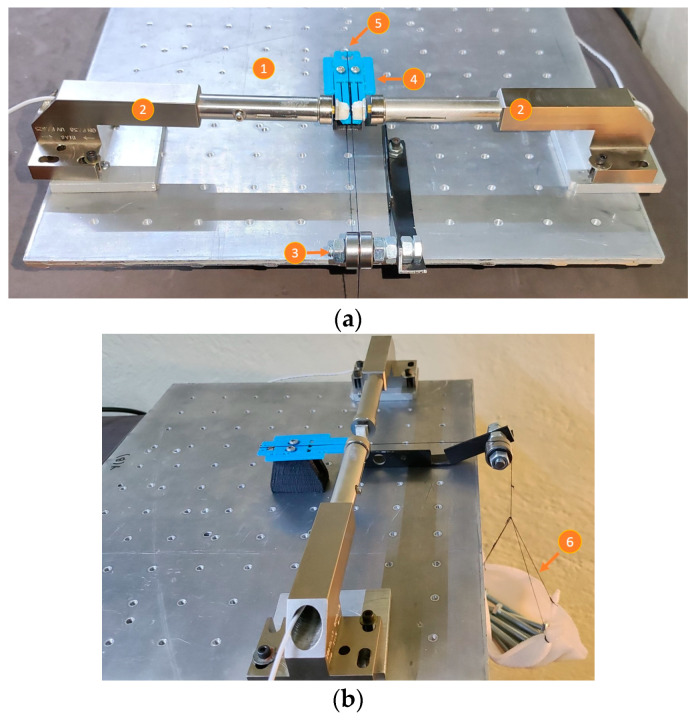
(**a**) Experimental setup to characterize the jaw displacement with the input force. (**b**) An applied load.

Once the amplification factor and the range of displacement at the input section of the MG are determined, it is necessary to know the magnitude of the force corresponding to this range. For this purpose, the experimental setup shown in Figure 15 was designed with the following elements:

1. Metallic base.
2. Inductive sensors (to measure output displacements).
3. Pulley formed by an arrangement of 2 bearings (to support the loads, reducing the friction).
4. MG.
5. Connecting wire (thread), which allows the connection between the load and the input of the MG.
6. Load (or proof mass).

For this test, equal screws were used to determine the force at the input stage (bridge-type mechanism) of the MG, with average weights of 11.82 g, from 1 to 12 elements, used. Figure 15 shows some screws that generate a force in the negative direction of the Z-axes, which can be calculated by Newton’s second law. The set of screws is suspended by means of a thread that passes through a pulley and is attached to the MG input. If the friction in the pulley and in the parts touched by the thread is neglected, it can be considered that the force in the direction of the ground is equal to the force at the input of the MG.

**Figure 16:**
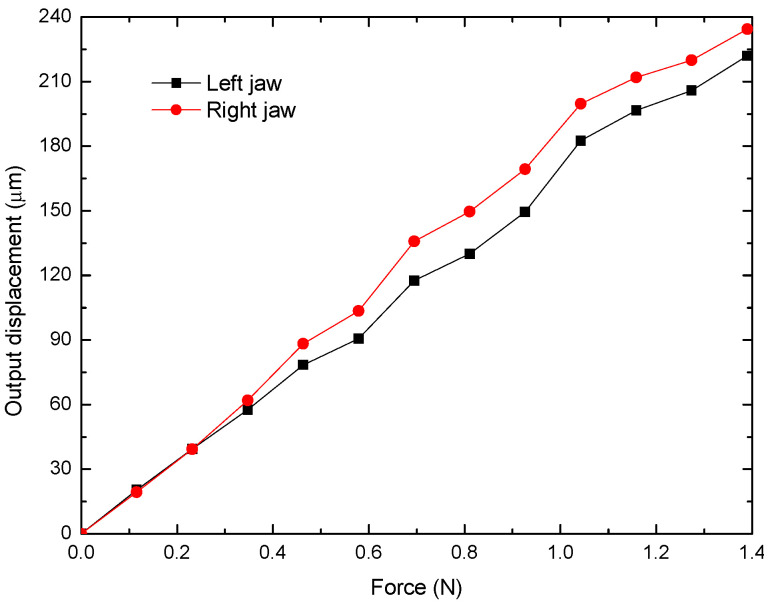
Output displacement of the jaws against the input force.

The displacement generated in the MG jaws has a linear trend, as can be observed in Figure 16. The right jaw has a larger displacement, with a maximum difference of 11.75%. Because the joining surface in the bridge mechanism is practically punctual, it can be said that the main reason for the asymmetry is the precision in the manufacturing process. The difference in the widths of the thinner manufactured parts may also be a determining factor.

**Figure 17:**
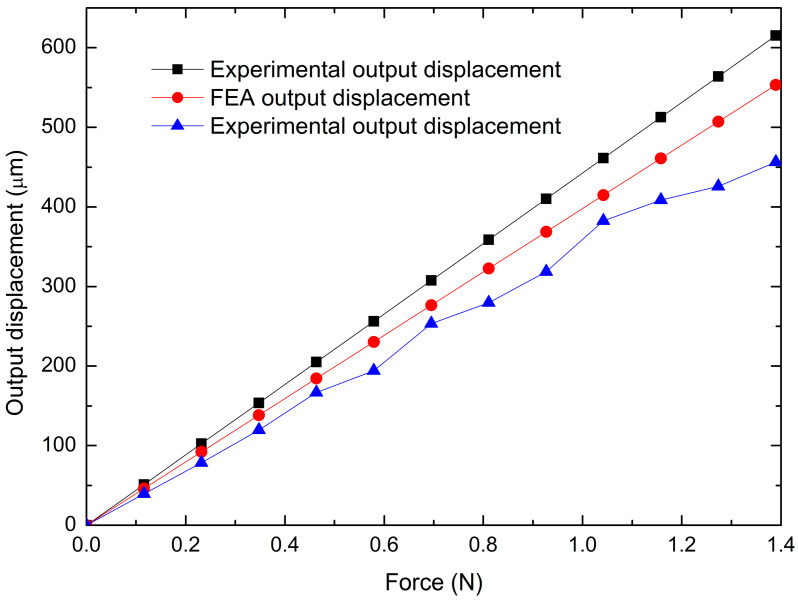
Comparison of the total output displacement of the MG, obtained from experimental tests and theoretical and FEA results.

Figure 17 shows a comparison of the experimental, theoretical, and FEA results for the MG output displacement. The trends in all cases tend to be linear. It should be noted that the displacements of the jaws are larger in the case of FEA, as expected. The additive manufacture has limitations in terms of the finishing accuracy of the 3D-printed structures; in addition, undesirable buckling of some of the large-sized levers may affect the maximum displacement of the MG jaws, thus deviating the experimental performance from that predicted by the numerical analysis. 

Figure 14 and Figure 17 show a comparison of the output displacement values obtained by theoretical, analytical, and experimental approximations in the following two cases:

- Input force (static analysis). Considering the FEA output displacement values as references, the maximum errors are 11.22% and 17.49%, respectively. The minimum errors are 11.21% and 13.88%, respectively.
- Input displacement (kinematic analysis). The comparison with the corresponding FEA values as references gives the maximum errors of 21.78% and 11.47%, respectively, while the minimums values are 21.59% and 2.66%, respectively.

With the experimental setups shown in this section, the ranges of input displacement (from 10 µm up to 70 µm) and input force (from 0.11 N up to 1.38 N) were obtained. For this characterization, the piezoelectric actuator was not used due to the nonlinear performance of its output variables (force and stroke).

### 6.4. Electric Operation of the Miniature Gripper

**Figure 18:**
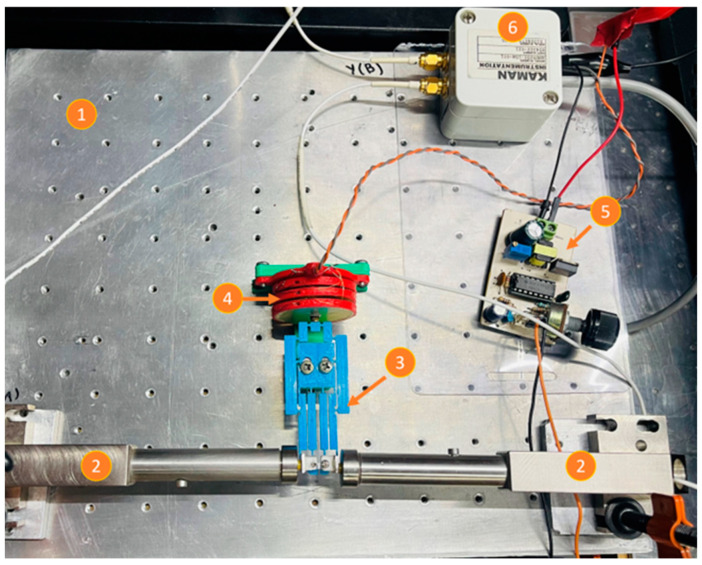
Experimental setup to characterize the piezoelectric stack-driven MG.

The behavior of the MG when the piezoelectric stack is applied at its input is analyzed in this section. The operating range of the stack actuator must be determined, depending on the displacement and force provided when it is fed. One disadvantage of this actuator is the hysteresis effect, which is inherent to its piezoelectric components. For this test, the experimental arrangement shown in Figure 18 was used, consisting of:

1. Metallic base.
2. Inductive sensors (to measure output stroke).
3. MG.
4. Piezoelectric stack.
5. Piezoelectric actuator driver.
6. Sensor control module.

**Figure 19:**
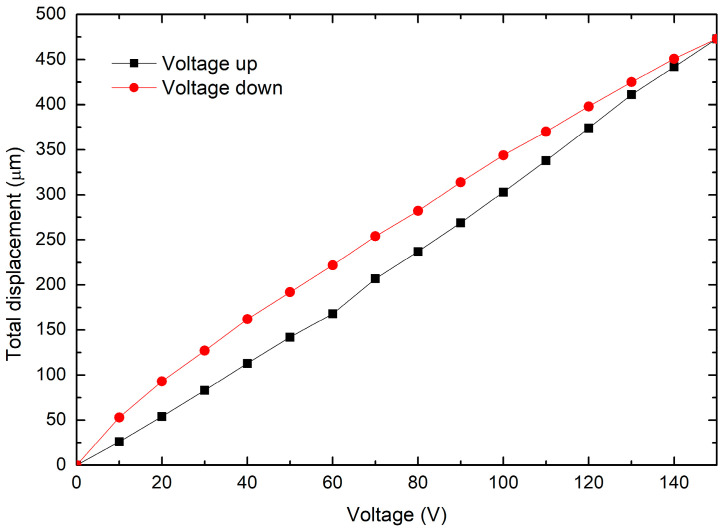
Total output displacement.

The total output displacement obtained by applying voltage to the piezoelectric stack is shown in Figure 19. Again, a linear trend of the jaw’s displacement can be seen, as in the case when force was applied (Figure 16). The voltage range is adequate to guarantee the physical integrity of the MG, because it is close to the experimental value obtained in the previous section. Total displacement reaches a value of 473 µm at 150 V. Therefore, the value of the experimental amplification factor is 10.38. Hysteresis is also observed.

**Figure 20:**
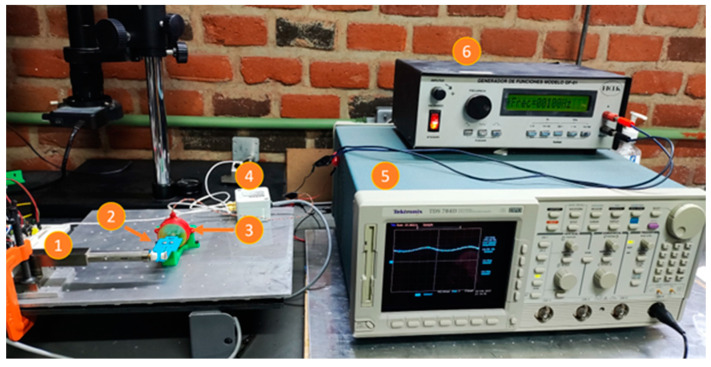
Experimental setup for the frequency test. 1. Inductive sensor. 2. Miniature gripper. 3. Piezoelectric stack. 4. Control module. 5. Oscilloscope Tektronik TDS 784D. 6. Frequency generator.

**Figure 21:**
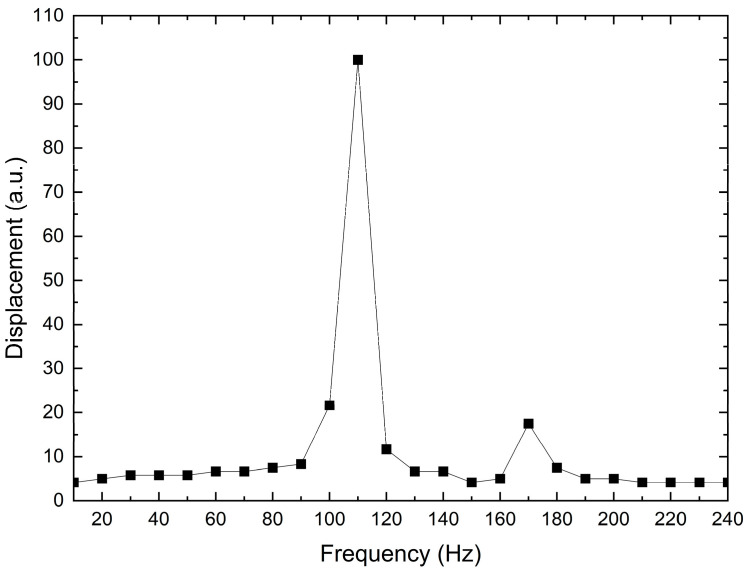
Graph of the frequency response of the piezoelectric-driven MG.

The experimental setup is given in Figure 20, and the results of the frequency test applied to the miniature gripper are shown in Figure 21. The procedure is similar to the one used for piezoelectric stack characterization. Two peaks in the graph of the frequency response of MG are reached at 110 Hz and 170 Hz, respectively. 

The first peak is near to the resonance frequency of the piezoelectric stack actuator (shown in Figure 11), of 120 Hz @ 20 V_pp_, with a difference of 10 Hz, equivalent to a percentage difference of 8.33%. The apparent reduction in the resonance frequency of the piezoelectric stack actuator can be attributed to the load represented by the MG. As is well-known, several types of excitations or loads can act on a vibrating system; in our case, the excitation is applied to the MG as input displacements, corresponding to the signal frequencies in the considered range, in steps of 10 Hz (inducing forced vibration) [55], which produces the opening/closing jaws. This can be assumed as the reason for the closeness between the first peak in the frequency response of the MG and the corresponding response of the piezoelectric stack. 

The second peak is closer to the fourth modal frequency, 192 Hz, with a percentual difference of 11.45%, which is an acceptable practical value. The coincidence of the directional excitation with the compliant mechanisms design performance supports this selective result. Some factors that may contribute to this error are the small differences between the dimensions of the flexural hinges, as well as the external forces applied to the MG, such as, in this case, undesirable vibration at fixed points or in the metal plate.

### 6.5. Operation Tests of the Miniature Gripper Holding Various Micro-Objects

With the position of the metallic pins used for this test, the initial aperture between the MG jaws is 500 µm, and the final aperture is 27 µm, implying that the appropriate diameters of the clamped objects could be in the range of 500 µm to 30 µm. 

Clamping tests of tiny objects such as salt grains, small parts of plants, electronic devices, and other objects made of dielectric or metallic materials, are shown in Figure S1 (part of the Supplementary Material). Larger sized objects or targets are shown in Figure S2. Video S1 about these tests can also be found as supplementary file. The weights of some representative samples are given in Table 6. Some of them are not given in Figures S1 and S2, such as the translucent ones.

These tests validate the performance of the MG as an effective tool for holding and clamping objects. The MG holds all elements under test firmly, despite the apparent small force of the piezoelectric stack and the fabrication process. Its use is convenient when a sample or object needs to be held for observation or manipulation over a relatively large period. Due to the operation mode of the MG, temperature is not a determinant parameter. However, it could be limited by the glass transition of PLA and the critical temperatures of the clamping objects.

Another advantage of the system proposed is the possibility to adjust and set the initial aperture of the MG. If it is required to clamp smaller objects, there are two possible ways to modify the opening range: the first one is to increase the mechanical preload by decreasing the distance between the anchors and the piezoelectric actuator, and the second one is to manually adjust the gap between the metal tips when attaching them to their corresponding support.

From the experimental tests, it is observed that it is desirable that the weights of the clamping objects would be less than 1350 mg, which corresponds to 13.24 mN. This value was chosen because it is in the range supported without exceeding the capacity of the MG. Other challenges are the object’s shape, due to the position of the mass center, and their texture. In our test, all objects were successfully clamped. The period of continuously clamping objects and the reliability of the MG depend on the absence of vibrations or air gusts. It is also necessary that the continuous power supply of the MG control system be stable, i.e., excessive heating may damage or cause failure of the MG response. For 15 continuous hours, no changes in the clamping process were obtained, using a resistor as proof mass.

### 6.6. Comparison with Other Microgrippers

The proposed MG was compared with representative piezoelectric-driven microgrippers made with aluminum (Table 7), obtained from Table 1. A microgripper with a similar geometry of the output and middle stages was also considered [56]. No additive manufactured and piezoelectric actuated miniature grippers have been found, only the previous design shown in [45]. 

Other grippers and microgrippers are made with soft materials, resins, etc., under different manufacturing processes such as stereolithographic and additive manufacturing, with thermal and magnetic actuation schemes, among others. Due to the different actuation schemes, it was not possible to make a comparison with them.

The proposed MG has the second largest displacement, being surpassed by [39]. However, it can be said that our proposal is competitive considering the material used. It should be noted that, comparing the proposed MG with all the aluminum ones, it has the lowest force, which can make it useful for clamping, holding, moving, and releasing low weight objects. The frequency is also the lowest, which limits its use for low frequencies. 

## 7. Conclusions

The proposal for a new microgripper is provided. It was additive manufactured, with PLA, which makes it a cost-effective design and eco-friendly. Aluminum is traditionally used for piezoelectric-driven devices; in this case, this material is used only in the tips to avoid the contact sections with the clamping objects, which could generate inconveniences due to the roughness generated by the 3D printing process. The devices used for the experimental characterization are of moderate cost, and several elements used in the corresponding setups were recovered from electronic waste. In general, the low-cost in manufacturing and implementation of the experimental and practical setups represent important advantages of the micromanipulation system developed.

In relation to its performance, structural changes were made to improve the design given in [45], all circular flexure hinges were homologated, and unnecessary material was eliminated in the bridge-type mechanism. In the parallelogram, oval flexure hinges were replaced by rectangular ones. With respect to the piezoelectric stack, the number and dimensions of buzzers was increased to generate a greater displacement without mechanical integrity risk for the MG structure.

In the experimental characterization, it was possible to establish the adequate power supply limits. The thinnest printed parts of the MG correspond to the flexure hinges, where the stress level is higher.

The characterization of the force to drive the input mechanism using various loads allowed us to obtain results very close to the theoretical ones, validating the experimental characterization. It is necessary to mention that the lack of accurate force measurement equipment was a limitation in the characterization tests. Therefore, an experimental arrangement was designed, where the repeatability of the results depends on the method used to add the proof masses (screws), because an abrupt placement would cause an oscillatory movement and consequently an unstable measurement of the sensors.

The maximum errors among theoretical, analytical, and experimental approximations for the output displacement in relation to the input force (kinematic analysis) of the MG, with the FEA values as the references, are 21.78% and 11.47%, respectively. In the case of the output displacement, considering the input force (static analysis), with the same reference source, the maximum errors are 11.22% and 17.49%, respectively. Linear trends of the output displacement are observed in both cases.

The piezoelectric stack consisting of 6 buzzers has a wide displacement range up to 367 µm without load. The hysteresis effect is present, like most piezoelectric drivers. It generates a low output force; however, for our purposes, this performance is enough. Due to the lower stiffness of the material from which this MG is made, compared to aluminum, which is commonly used in piezoelectrically actuated grippers, the designed actuator can drive the compliant mechanisms, achieving, despite the low amplification factor, generation of the second largest displacement when compared to the microgrippers shown in Table 7. The initial aperture of the MG is 500 μm, and it closes (jaws spacing) until 27 μm; this fact determines the diameter of the clamping objects. If the MG is required for subjection of smaller objects, there are two possible ways to modify the opening range. The first one is to increase the mechanical preload by decreasing the distance between the anchors and the piezoelectric actuator, with the support of the oval grooves. The second one is to determine the appropriate location of the metal jaws. This possibility of adjusting the initial opening is an advantage of this MG. The output force allows objects weighing less than 1.4 g to be clamped.

The operation of the MG has two limitations caused by the manufacturing material. The MG cannot operate where the environmental temperature is higher than 45 °C because this is the value where the glass transition temperature range of PLA begins [52]. In addition, compliant mechanisms made of PLA do not transmit sufficient force to allow the MG to cut objects, as in the case of aluminum. The proposed MG could be used together with a micropositioner with three DOFs previously shown in [8] to form a complete micromanipulation or microassembly system, where the MG could hold and release objects at specific locations, automatically. This complete system could reach up to six DOFs, making it capable of moving, clamping, and manipulating parts. When the MG would be assembled, it could open/close, rotate, and tilt (adding three DOFs to the system). This functional prototype also requires developing a real-time control and monitoring system. 

## Figures and Tables

**Table 2:** Configuration of the printing parameters with PLA in Ultimaker Cura© (version 4.1.0) used for the MG proposed.

Parameter	PLA
Layer Height	0.2 mm
Infill Density	100 %
Infill Pattern	Lines
Printing Temperature	200 °C
Build Plate Temperature	45–60 °C
Enable Retraction	Yes
Print Speed	60 mm/s
Enable Print Cooling	Yes
Build Plate Adhesion Type	Skirt

**Table 3:** Rigid linkage elements dimensions.

Letter	Dimensions (mm)
a	26.10
b	8.52
c	21.00
d	10.27
e	5.17
f	26.1

**Table 4:** Properties of PLA [51,52].

Parameter and Units	PLA
Young Modulus, (GPa)	3.5
Poisson’s Ratio	0.4
Tensile Strength, (MPa)	21–60
Ultimate tensile strength, (MPa)	73
Density, (kg/m^3^)	1250
Melting point, (°C)	145–177

**Table 5:** Technical details of FEA.

Device	Solver Target	Element Type/ Mesh	Inflation			Convergence		Total Mass (g)
			Transition Ratio	Max. Layers	Growth Rate	No. of Total Nodes	No. of Total Elements	
MG	Mechanical APDL	Triangle Surface Mesher/Program Controlled	0.272	5	1.2	21,096	10,253	1.89 *

NOTE: * Simulation of displacement, force, and stress were carried out without metallic pins.

**Table 6:** Weights of some objects.

Clamping Object	Weight (mg)
Fiber optics	6
Copper strip	11
Teflon	15
Kapton	22
Washer	177
Resistor	194
Nut with resistor	1342

**Table 7:** Comparison of the proposed MG with other grippers based on piezoelectric actuation.

Ref.	Year	Material	Amplification Factor	Output Maximum Displacement (µm)	Output Force (mN)	Frequency (Hz)
[12]	2015	AL7075T651	22.8	190	1000	N.A.
[39]	2015	AL7075	21.4	427.8	50	N.A.
[41]	2021	Mn65 and Al-7075	52.8	918	50	217.2
[45]	2019	PLA	6.5	382	8.84	N.A.
[56]	2020	AL7075-T6	12.76	93.52	N.A.	1044
This proposal	2023	PLA	10.38	473	13.24	93.3

## Supplementary Materials

The following supporting information can be downloaded at: https://www.mdpi.com/article/10.3390/mi14040727/s1, Figure S1: Microgripper holding tiny and lightly objects; Figure S2: Microgripper clamping several small objects; Video S1: Clamping tests with different objects.
