# Supplementary material for: Additive Manufactured Piezoelectric-Driven Miniature Gripper
Source: Micromachines (Basel). 2023 Mar 25;14(4):727. doi: 10.3390/mi14040727 (PMC10141374; doi:10.3390/mi14040727)
Supplement: Supplementary file 1 [file micromachines-14-00727-s001.zip › micromachines-2272585-supplementary.pdf]

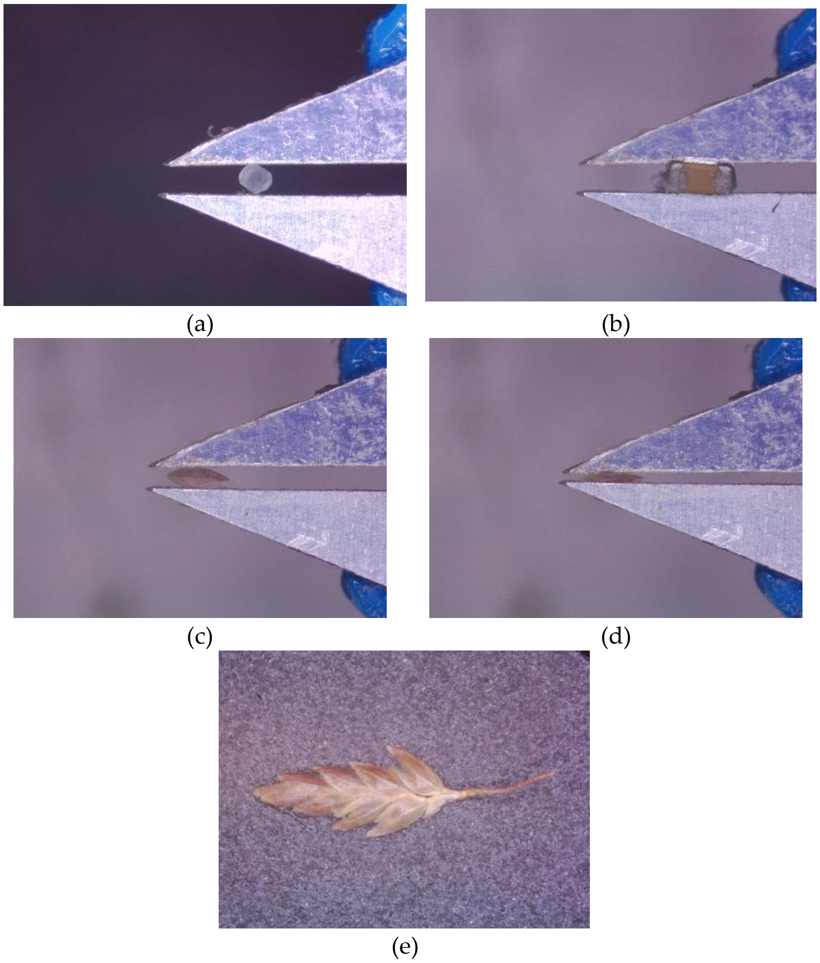

Figure S1. Microgripper holding tiny and lightly objects. (a) One salt grain. (b) Surface mount technology (SMD) capacitor. (c) Little seed with jaws aperture of 400 μm, and (d) 170 μm. (e) Image of the little branch from which the little seed was taken.

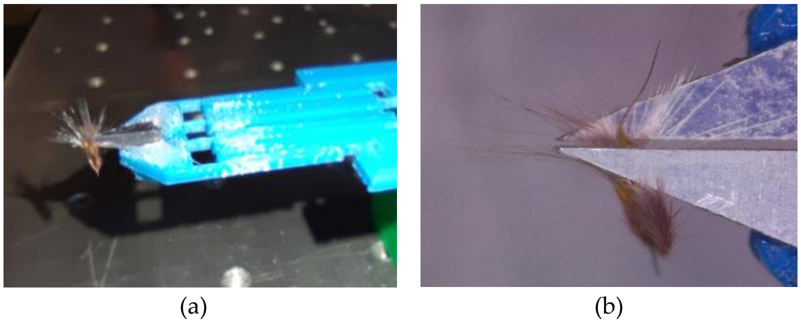

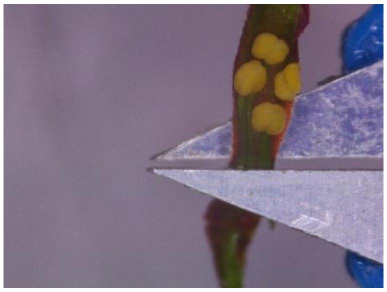
(c)

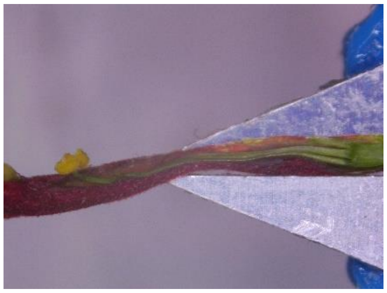
(d)

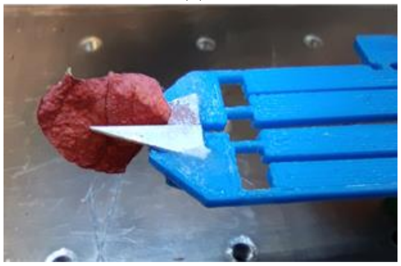
(e)

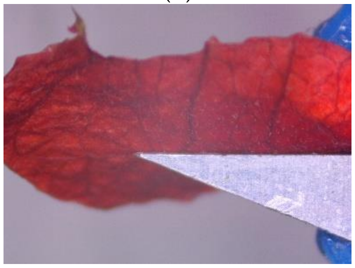
(f)

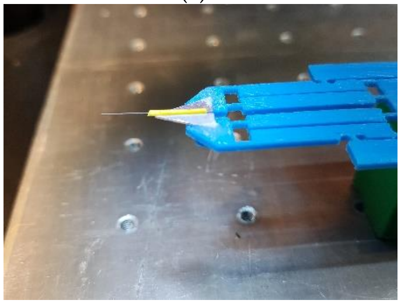
(g)

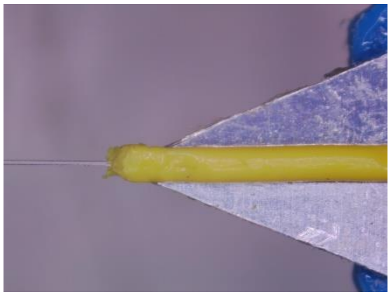
(h)

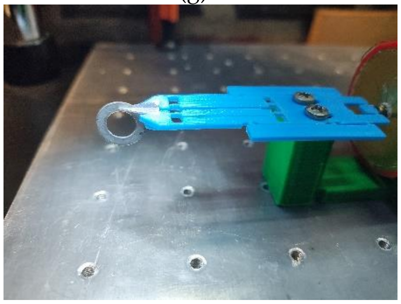
(i)

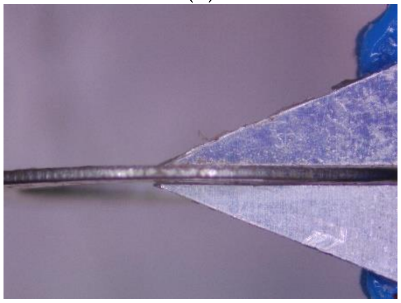
(j)

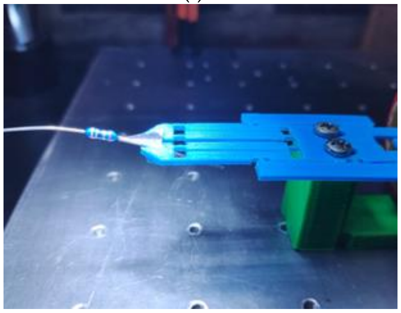
(k)

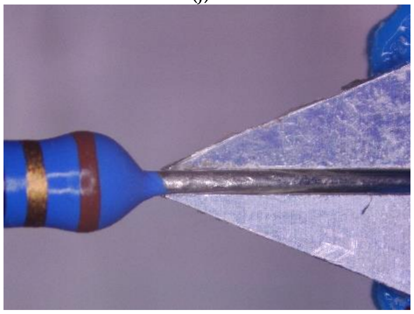
(l)

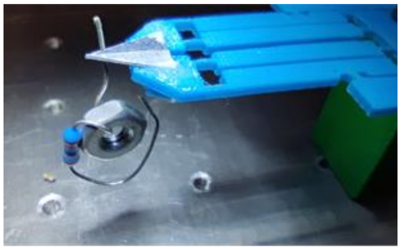

(m)

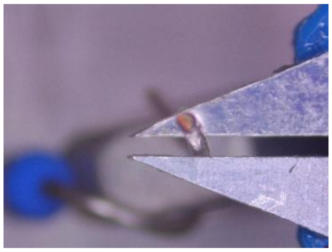

(n)

Figure S2. Microgripper clamping several small objects, normal and zoom-in views of: (a) and (b) a little flower, (c) and (d) stigma transversal section, (e) and (f) bougainvillea petal, (g) and (h) optical fiber section, (i) and (j) washer, (k) and (l) resistor, (m) and (n) nut and resistor.
